# Comparing genotypes and antibiotic resistance profiles of *Mycobacterium abscessus* and *Mycobacterium massiliense* clinical isolates in China

**DOI:** 10.1017/S0950268821002211

**Published:** 2021-10-11

**Authors:** Yiting Wang, Wencong He, Ping He, Huiwen Zheng, Yanlin Zhao

**Affiliations:** 1 National Tuberculosis Reference Laboratory, Chinese Center for Disease Control and Prevention, Beijing 102200, China; 2 Planned Immunity Inoculation Institute, Beijing Center for Disease Prevention and Control, Beijing, China; 3 Laboratory of Respiratory Diseases, Beijing Key Laboratory of Pediatric Respiratory Infection Diseases, Beijing Pediatric Research Institute, Beijing Children's Hospital, Capital Medical University, Key Laboratory of Major Diseases in Children, Ministry of Education, National Clinical Research Center for Respiratory Diseases, National Center for Children's Health, Beijing 100045, China

**Keywords:** Drug susceptibility testing, genotype, nontuberculous mycobacteria

## Abstract

This study aimed to investigate differences in the antimicrobial susceptibility of members of the *Mycobacterium abscessus* complex (MABC): subsp. *massiliense and* subsp. *abscessus*, and to identify associations between strain genotypes and antimicrobial resistance phenotypes. A total of 383 clinical MABC isolates (subsp. *abscessus*: *n* = 218, 56.9%; subsp. *massiliense*: *n* = 163, 42.6%; subsp. *bolletii*: *n* = 2, 0.5%) were characterised using multilocus variable number tandem repeat (VNTR) typing and drug susceptibility testing. Most isolates exhibited susceptibility to amikacin, clarithromycin and azithromycin but resistance to cefoxitin and minocycline was statistically more associated with isolates unclustered by VNTR type. The Simpson's diversity indexes of VNTR typing for *M. abscessus* and *M. massiliense* isolates were 0.999 and 0.997, respectively. Genotyping of *M. abscessus* and *M. massiliense* isolates by VNTR may provide valuable information for predicting resistance phenotype.

## Introduction

Nontuberculous mycobacteria (NTM) have been increasingly recognised as causative agents of opportunistic infections of humans, generally affecting the lungs, skin and soft tissue and visceral or disseminated in severely immunosuppressed individuals [1]. The organisms are generally classified according to their rapid, or slow, rates of growth on culture media [2]. *Mycobacterium abscessus* is the most frequently encountered rapid grower among NTM causing pulmonary infections, and accounts for 65–80% of cases [3]. The group is characterised by extensive antimicrobial drug resistance which often results in unsatisfactory clinical treatment outcomes [4].

*M. abscessus* complex (MABC) isolates have been further differentiated on the basis of *rpoB* sequences into three closely related subspecies, namely: *M. abscessus*, *Mycobacterium massiliense* and *M. bolletii* [5]. In spite of several shared taxonomic characteristics, the subspecies exhibit distinctly different antibiotic resistance phenotypes and treatment outcomes [6]. Differences are particularly pronounced between *M. abscessus* and *M. massiliense*, especially in the treatment response rates of patients to clarithromycin (CLA)-based antibiotic therapy, where greater efficacy was evident for pulmonary patients infected with *M. massiliense* than with *M. abscessus* [2, 7]. Similarly, *M. massiliense* was reported to have greater susceptibility to doxycycline, although this was based on small numbers of *M. abscessus* isolates tested [2]. Nevertheless, since these results suggested that subspecies and possibly strain genotypes, may be predictive of antibiotic resistance phenotype, further testing of this concept was conducted in this larger study.

Variable number tandem repeat (VNTR) analysis has been used as a suitable tool for genetic fingerprinting of several bacterial species, including the pathogenic mycobacteria [8]. Indeed, a recent study revealed that specific VNTR genotypes were associated with drug resistance in isolates of both *M. tuberculosis* and *M. intracellulare* [9]. However, an association of drug resistance profiles within the *M. abscessus* complex has not to our knowledge been previously described.

In this study, isolates of the species complex were characterised to the subspecies level and correlations of drug resistant profiles with subspecies analysed, with the ultimate goal of identifying predictive associations between strain genotypes and drug resistance phenotypes.

## Methods

### Clinical isolates and species identification

In total, 383 MABC isolates were recovered from clinical specimens collected between 2014 and 2016 from patients in two specialised TB treatment hospitals in China. All patients providing isolates met the microbiological and clinical criteria of the American Thoracic Society for a diagnosis of NTM pulmonary disease [1]. The study was approved by the Chinese Center for Disease Control and Prevention.

Clinical samples were cultured on Löwenstein–Jensen (L–J) medium after alkali treatment with 4% NaOH. After 4 days of incubation, colonies were scraped from the surface of the medium for the preparation of genomic DNA as previously reported [10]. Identification of subspecies was determined by multilocus sequence typing of genes encoding 16S rRNA, *hsp65*, *rpoB* and 16S-23S rRNA internal transcribed spacer sequence loci [11].

### Antimicrobial susceptibility testing

The drug susceptibility profiles of isolates were determined by a broth microdilution method according to the guidelines of the Clinical and Laboratory Standards Institute (CLSI) [12]. Briefly, freshly grown colonies from L–J slants were vortexed with glass beads in saline. The resulting suspension was adjusted to 0.5 McFarland and further diluted 1:200 in cation-adjusted Mueller–Hinton broth before being added to 96-well microtitre plates containing serial doubling-dilutions of antimicrobial agents. After incubation at 37 °C for 72 h, the minimum inhibitory concentration (MIC) was defined as the lowest concentration of antibiotic to inhibit visible growth of mycobacteria.

The following 18 antimicrobial agents in a concentration range of 0.0625–256 μg/ml were tested: clarithromycin (CLA), amikacin (AMK), moxifloxacin (MXF), linezolid (LZD), rifabutin (RFB), tobramycin (TOB), meropenem (MEM), imipenem (IMP), cefoxitin (FOX), capreomycin (CAP), azithromycin (AZM), levofloxacin (LVX), gatifloxacin (GAT), minocycline (MNO), tigecycline (TIG), sulphamethoxazole (SFX), streptomycin (STR) and clofazimine (CFM). Classification of susceptibility was recorded according to CLSI breakpoints, and where unavailable, was compared with previously published studies [9, 13, 14].

#### VNTR analysis

VNTR typing of isolates was performed by the method of Wong *et al*. [15], but with the exclusion of locus TR20 due to amplification failure; analysis was therefore based on 17 loci. The discriminatory power of VNTR typing was calculated using Simpson's index of diversity as previously described [16]. Clustered strains were defined as isolates with the same genotype, compared with ‘unclustered’ isolates of different genotypes.

## Results

### Antimicrobial susceptibility

Isolates were identified to the subspecies level as *M. abscessus* (218, 56.9%), *M. massiliense* (163, 42.6%) and *M. bolletii* (2, 0.5%). The results of antimicrobial susceptibility testing grouped according to MIC ranges are summarised in Table 1. The three most effective agents against both *M. abscessu*s and *M. massiliense* isolates were AMK, CLA and AZM, with respective resistance rates for *M. abscessus* of 2.8%, 6.4% and 15.1%, and for *M. massiliense* of 4.3%, 10.4% and 12.9%; no significant differences in resistance to these drugs were observed between subspecies (*P* = 0.383, 0.151 and 0.535, respectively). LZD and FOX each exhibited activity against a moderate number of *M. abscessus* and *M. massiliense* isolates, with respective species resistance rates to LZD of 15.6% *vs.* 22.1%, and to FOX of 25.7% *vs.* 31.3%, with no statistical difference between species observed (*P* > 0.05). With regards to MXF, GAT, SFX and TIG results, antibiotic resistance rates were lower for *M. abscessus vs. M. massiliense* as follows: 55.0% *vs.* 66.3%, *P* = 0.016 for MXF; 45.4% *vs.* 62.6%, *P* < 0.001 for GAT; 53.7% *vs.* 76.7%, *P* < 0.001 for SFX and 22.9% *vs.* 34.3%, *P* < 0.001 for TIG. In contrast, a greater percentage of *M. abscessus* isolates were resistant to MNO (198, 90.8%), compared with 50.9% of *M. massiliense* (*P* < 0.001).

**Table 1.** MICs (μg/ml) of antimicrobials against *M. abscessus* and *M. massiliense* isolates

Drug	Species	MIC_50_^a^	MIC_90_	No. of isolates^b^ (%)			*P* value
				Susceptible	Intermediate	Resistant	
CLA	*M. abscessus*	0.25	8	196 (89.9)	8 (3.7)	14 (6.4)	0.151
	*M. massiliense*	0.125	16	139 (85.3)	7 (4.3)	17 (10.4)	
AMK	*M. abscessus*	8	16	203 (93.1)	9 (4.1)	6 (2.8)	0.383
	*M. massiliense*	16	64	145 (89.0)	11 (6.7)	7 (4.3)	
MXF	*M. abscessus*	8	16	35 (16.1)	63 (28.9)	120 (55.0)	0.016
	*M. massiliense*	8	16	14 (8.6)	41 (25.2)	108 (66.3)	
LZD	*M. abscessus*	16	64	154 (70.6)	30 (13.8)	34 (15.6)	0.095
	*M. massiliense*	16	64	104 (63.8)	23 (14.1)	36 (22.1)	
TOB	*M. abscessus*	16	32	14 (6.4)	69 (31.7)	135 (61.9)	0.028
	*M. massiliense*	32	64	5 (3.1)	10 (6.1)	148 (90.8)	
MEM	*M. abscessus*	128	256	1 (0.5)	4 (1.8)	213 (97.7)	0.195
	*M. massiliense*	256	256	3 (1.8)	1 (0.7)	159 (97.5)	
IMP	*M. abscessus*	128	256	3 (1.4)	34 (15.6)	181 (83.0)	0.963
	*M. massiliense*	128	256	2 (1.2)	35 (21.5)	126 (77.3)	
FOX	*M. abscessus*	128	256	13 (6.0)	149 (68.3)	56 (25.7)	0.715
	*M. massiliense*	128	256	10 (6.1)	102 (62.6)	51 (31.3)	
LVX	*M. abscessus*	16	32	5 (2.3)	7 (3.2)	206 (94.5)	0.928
	*M. massiliense*	16	32	4 (2.5)	4 (2.5)	155 (95.0)	
GAT	*M. abscessus*	4	16	45 (20.6)	74 (33.9)	99 (45.4)	<0.001
	*M. massiliense*	8	16	15 (9.2)	46 (28.2)	102 (62.6)	
MNO	*M. abscessus*	64	256	10 (4.6)	10 (4.6)	198 (90.8)	<0.001
	*M. massiliense*	16	64	54 (33.1)	26 (16.0)	83 (50.9)	
TIG	*M. abscessus*	4	16	68 (31.2)	100 (45.9)	50 (22.9)	<0.001
	*M. massiliense*	8	64	21 (12.9)	86 (52.8)	56 (34.3)	
SFX	*M. abscessus*	128	256	101 (46.3)	–	117 (53.7)	<0.001
	*M. massiliense*	128	256	38 (23.3)	–	125 (76.7)	
STR	*M. abscessus*	64	64	5 (2.3)	–	213 (97.7)	0.443
	*M. massiliense*	64	64	2 (1.2)	–	161 (98.8)	
CFM	*M. abscessus*	1	8	131 (60.1)	–	87 (39.9)	0.057
	*M. massiliense*	1	64	82 (50.3)	–	81 (49.7)	
RFB	*M. abscessus*	8	16	6 (2.8)	–	212 (97.2)	0.169
	*M. massiliense*	8	32	9 (5.5)	–	154 (94.5)	
CAP	*M. abscessus*	32	64	8 (3.7)	–	210 (96.3)	0.756
	*M. massiliense*	32	64	7 (4.3)	–	156 (95.7)	
AZM	*M. abscessus*	8	64	185 (84.9)	–	33 (15.1)	0.533
	*M. massiliense*	2	64	142 (87.1)	–	21 (12.9)	

a MIC_50_ represents the concentration required to inhibit the growth of 50% of the strains; MIC_90_ represents the concentration required to inhibit the growth of 90% of the strains.

b Susceptibility and resistance breakpoints as recommended by the Clinical and Laboratory Standards Institute (CLSI-M24-A2) [12].

### Genotyping and clustering analysis

**Fig. 1.**
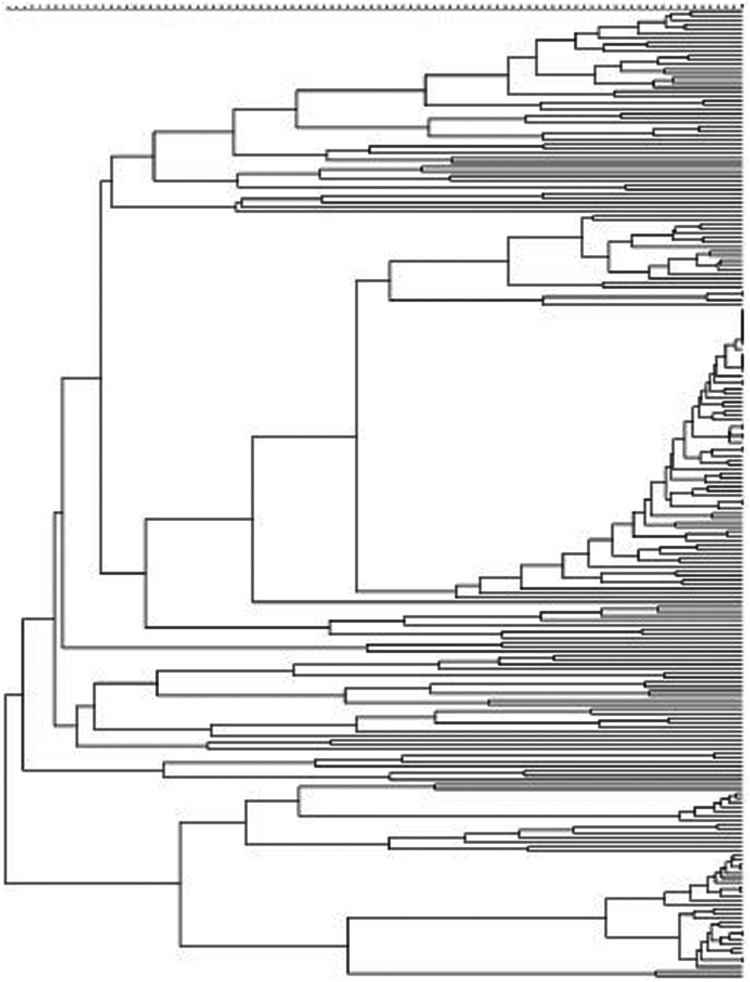
Phylogenetic tree of *M. abscessus*

**Fig. 2.**
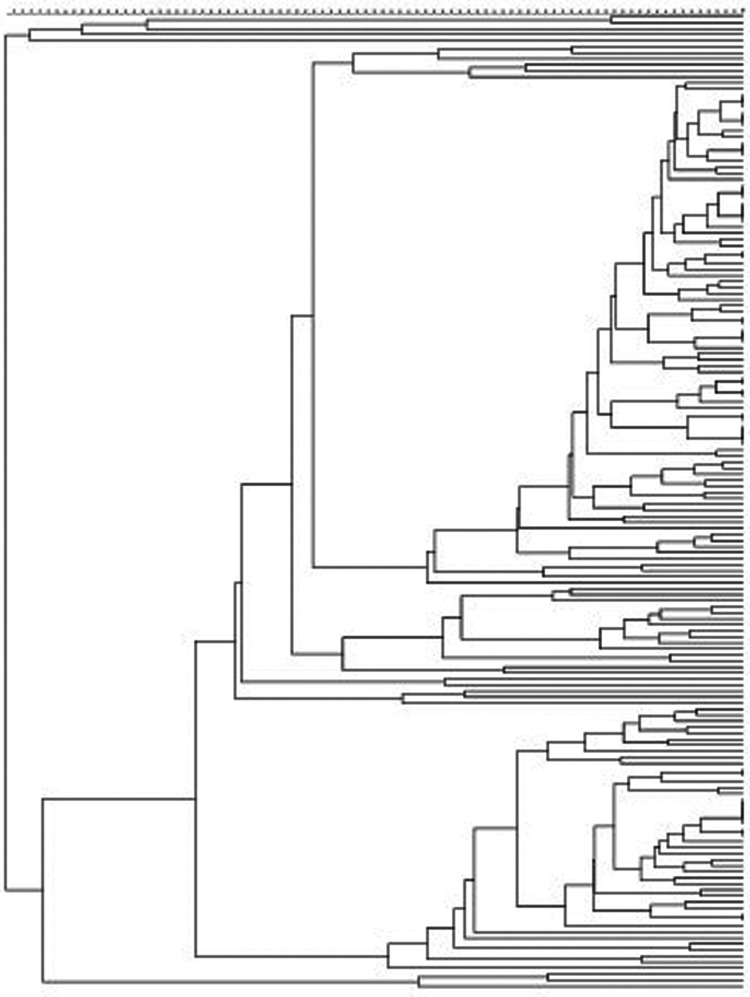
Phylogenetic tree of *M. massiliense*

**Fig. 3.**
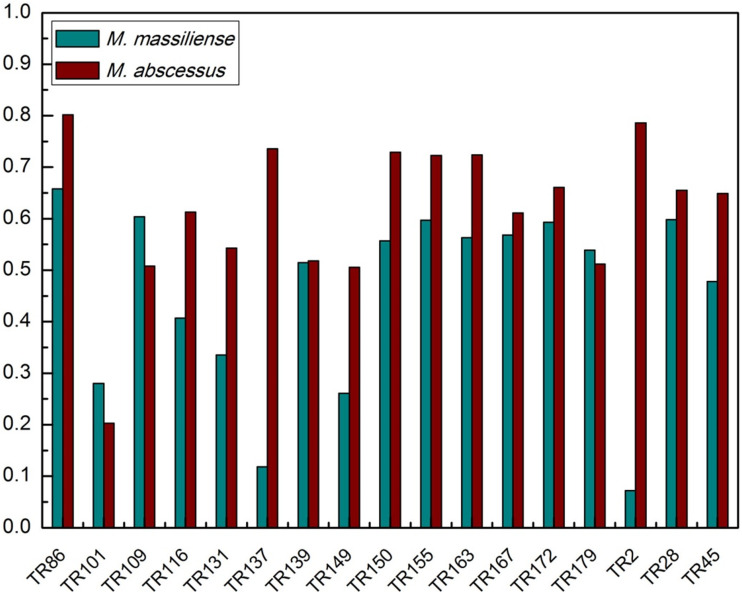
VNTR allelic distribution in *M. abscessus* and *M. massiliense* clinical isolates

All isolates, with the exception of the two *M. bolletii*, were genotyped by VNTR and assigned to phylogenetic clusters. The 218 *M. abscessus* representatives were differentiated into 10 clusters (comprising two to nine isolates per cluster) and 188 unique genotypes (Fig. 1). Similarly, 119 of the 163 *M. massiliense* isolates were differentiated into unique genotypes, with the remaining 44 isolates grouped into 16 clusters (two to five isolates per cluster) (Fig. 2). The clustering rate of *M. massiliense* was 17.2%, compared with 9.2% for *M. abscessus* (*P* > 0.05). Analysis of the allelic diversity of VNTR loci between *M. abscessus* and *M. massiliense* (Fig. 3) isolates showed that most *M. abscessus* exhibited allelic diversity, with the exception of locus TR101. Notably, loci TR2 and TR137 were found to be less polymorphic and thus less discriminatory between *M. massiliense* isolates (*h* < 0.2), whereas the opposite was true for *M. abscessus* (*h* > 0.6).

### VNTR genotype and drug susceptibility

Associations between VNTR genotype and drug susceptibility profiles of *M. abscessus* and *M. massiliense* (Table 2) showed that of the 188 unclustered isolates of the former, 53 (28.2%) were resistant to FOX which was a significantly higher rate than that for clustered isolates (3/30, 10.0%, *P* = 0.041). Similarly, for *M. massiliense*, the rate of MNO resistance among unclustered isolates (65/119, 54.6%) was significantly higher than among clustered isolates (18/44, 40.9%, *P* = 0.029). No significant differences were evident for other drug resistance rates between clustered and unclustered isolates within *M. abscessus* or *M. massiliense* groups of isolates (*P* > 0.05) (Supplementary table).

**Table 2.** Susceptibility to cefoxitin (FOX) and minocycline (MNO) of clustered and unclustered strains of *M. abscessus* and *M. massiliense*

Drug	Species	Strain class	No. susceptible (%)	No. resistant (%)	*X*^2^	*P*
FOX	*M. abscessus*	Clustered	3 (10.0)	3 (10.0)	4.173	0.041
		Unclustered	10 (5.3)	53 (28.2)		
	*M. massiliense*	Clustered	5 (11.4)	14 (31.8)	1.982	0.159
		Unclustered	5 (4.2)	37 (31.1)		
MNO	*M. abscessus*	Clustered	0 (0.0)	30 (100.0)	1.771	0.183
		Unclustered	10 (5.3)	168 (89.4)		
	*M. massiliense*	Clustered	21 (47.7)	18 (40.9)	4.754	0.029
		Unclustered	33 (27.7)	65 (54.6)		

## Discussion

Rapidly growing mycobacteria have in recent years been increasingly implicated as causes of extensive pulmonary infections in many countries [17, 18]. Due to their natural resistance to most commonly administered antibiotics, infections by MABC members, especially the prevalent subspecies *M. abscessus*, requires special treatment regimens [6, 7]. In this study, we compared *in vitro* drug susceptibility profiles between isolates of these subspecies from patients in China, and explored potential associations of antimicrobial susceptibility and strain genotypes that might have predictive value for guiding antimicrobial therapy.

The key finding was that both antibiotic susceptibility profiles and genotyping results differed between *M. abscessus* and *M. massiliense*. Of the three cornerstone drugs CLA, AMK and FOX, used for treatment of *M. abscessus* infections, CLA and AMK exhibited the highest activities against both species, but with a higher percentage of CLA-resistant strains of *M. massiliense* than *M. abscessus*, as was also observed by Nie *et al*. [19]. However, according to Harada *et al*. [20], CLA resistance rates of *M. abscessus* eclipsed those for *M. massiliense* in experiments based on a 14-day incubation period rather than the conventional 3-day period. Nevertheless, our results are in agreement with Nie *et al*. [19] in that resistance in *M. massiliense* is more extensive than in *M. abscessus* in China. We found high resistance rates to MXF, GAT and LVX in both subspecies, particularly for LVX which was much higher than those for MXF and GAT. These findings are consistent with previous reports that MABC isolates are frequently fluoroquinolone-resistant [19]. This phenomenon may be due to the overuse of fluoroquinolones in China, although some isolates are naturally resistant to this antimicrobial class [19]. Moreover, resistant rates to SFX and TIG among *M. abscessus* were shown here to be significantly lower than that for *M. massiliense*, indicating increased resistance in the latter. By contrast, *M. massiliense* proved to be more susceptible to MNO compared to *M. abscessus*, a finding in line with Adekambi *et al*. [21].

VNTR typing based on 17-loci was highly discriminatory for the two subspecies with DI（Diversity Index）values of 0.999 and 0.997, respectively, slightly exceeding values obtained for Malaysian and Japanese isolates [15, 22]. Of differences in TR-2 and -137 loci (DI, respectively, 0.400 and 0.392 in Malaysia, and 0.786 and 0.736 in China), allelic diversity among most other *M. abscessus* loci was similar to previously published data [15]. This was speculated to be possibly due to phylogenetic incongruence among strains from different geographic regions, resulting in alterations in the discriminatory power of some loci. Most VNTR loci, except for TR101, of *M. massiliense* showed less allelic diversity than for *M. abscessus*, which suggests that these loci might be more suitable for differentiation between isolates of the latter. Similarly, the higher cluster rate of *M. massiliense* isolates may be a reflection related to pathogenicity associated with the severity of the epidemic, a concept which warrants further exploration.

To our knowledge, there are no comparable reports from China which have investigated strain VNTR genotype associations with drug resistance phenotypes for *M. abscessus* and *M. massiliense* on such a large number of samples. We observed that FOX resistance was more common among unclustered than clustered strains of *M. abscessus*, the former being significantly associated with MNO resistance in *M. massiliense*. Although NTM are regarded as opportunists that cause infections in both immunocompromised and vulnerable immunocompetent hosts [23], the observed resistance to FOX and MNO may be related to the pathogenicity of MABC strains and host preferences, leading to a biased distribution of these resistances among the subspecies.

Our study had two key limitations. First, we did not analyse *M. bolletii* isolates, due to the small number of samples, and second, as had been done in several other studies, we used 37 °C instead of 30 °C for DST (Drug susceptibility testing) analysis of rapid growers.

In conclusion, these data illustrate that among MABC isolates in China, *M. abscessus* was the most common subspecies followed by *M. massiliense*. AMK, CLA and AZM exhibited high antimicrobial activities but with significantly different resistance rates to MXF, GAT, SFX, TIG and MNO, between subspecies. Additionally, 17-loci VNTR typing had high discriminatory power for differentiation of clustered and unclustered isolates. Given the wide heterogeneity of resistance profiles to currently available antimicrobials, further genotype differentiation among subspecies may prove to be predictive of antibiotic resistance phenotypes, and inform more effective antibiotic regimens to optimise treatment outcomes for patients suffering from NTM pulmonary diseases.

## Data Availability

All data generated or analysed during this study are included in this published article.

## Supplementary material

For supplementary material accompanying this paper visit http://dx.doi.org/10.1017/S0950268821002211. click here to view supplementary material
