# Supplementary material for: Comparing genotypes and antibiotic resistance profiles of Mycobacterium abscessus and Mycobacterium massiliense clinical isolates in China
Source: Epidemiol Infect. 2021 Oct 11;149:e232. doi: 10.1017/S0950268821002211 (PMC8569833; doi:10.1017/S0950268821002211)
Supplement: Supplementary file 1 [file S0950268821002211sup001.docx]

**Supplemental table 1**

Drug susceptibility profiles of clustered and unclustered strains of *M. abscessus* and *M.massiliense* isolates

| Drug | species | Strain class | No.(%) of Sensitive strain | No.(%) of Resistant strain | *X^2^* | *P* |
| --- | --- | --- | --- | --- | --- | --- |
| CLA | *M.abscessus* | clustered | 27 (90.0) | 3 (10.0) | 0.625 | 0.429 |
|  |  | unclustered | 169 (89.9) | 11 (5.9) |  |  |
|  | *M.massiliense* | clustered | 42 (95.5) | 2 (4.5) | 2.547 | 0.111 |
|  |  | unclustered | 97 (81.5) | 15 (12.6) |  |  |
| AMK | *M abscessus* | clustered | 30 (100.0) | 0 (0.0) | 1.035 | 0.309 |
|  |  | unclustered | 173 (92.0) | 6 (3.2) |  |  |
|  | *M.massiliense* | clustered | 41 (93.2) | 2 (4.5) | 0.000 | 0.986 |
|  |  | unclustered | 104 (87.4) | 5 (4.2) |  |  |
| MXF | *M.abscessus* | clustered | 6 (20.0) | 16 (53.3) | 0.323 | 0.570 |
|  |  | unclustered | 29 (15.4) | 104 (55.3) |  |  |
|  | *M.massiliense* | clustered | 4 (9.1) | 29 (65.9) | 0.019 | 0.892 |
|  |  | unclustered | 10 (8.4) | 79 (66.4) |  |  |
| LZD | *M.abscessus* | clustered | 21 (70.0) | 6 (20.0) | 0.364 | 0.546 |
|  |  | unclustered | 133 (70.7) | 28 (14.9) |  |  |
|  | *M.massiliense* | clustered | 31 (70.5) | 9 (20.5) | 0.303 | 0.582 |
|  |  | unclustered | 73 (61.3) | 27 (22.7) |  |  |
| RFB | *M.abscessus* | clustered | 0 (0.0) | 30 (100.0) | 0.985 | 0.321 |
|  |  | unclustered | 6 (3.2) | 182 (96.8) |  |  |
|  | *M.massiliense* | clustered | 1 (2.3) | 43 (97.7) | 1.219 | 0.269 |
|  |  | unclustered | 8 (6.7) | 111 (93.3) |  |  |
| TOB | *M.abscessus* | clustered | 2 (6.7) | 13 (43.3) | 0.304 | 0.582 |
|  |  | unclustered | 12 (6.4) | 122 (64.9) |  |  |
|  | *M.massiliense* | clustered | 1 (2.3) | 41 (93.2) | 0.144 | 0.704 |
|  |  | unclustered | 4 (3.4) | 107 (89.9) |  |  |
| MEM | *M.abscessus* | clustered | 0 (0.0) | 30 (100.0) | 0.164 | 0.686 |
|  |  | unclustered | 1 (0.5) | 183 (97.3) |  |  |
|  | *M.massiliense* | clustered | 1 (2.3) | 43 (97.7) | 0.059 | 0.808 |
|  |  | unclustered | 2 (1.7) | 116 (97.5) |  |  |
| IMP | *M.abscessus* | clustered | 1 (3.3) | 24 (80.0) | 1.013 | 0.314 |
|  |  | unclustered | 2 (1.1) | 157 (83.5) |  |  |
|  | *M.massiliense* | clustered | 1 (2.3) | 32 (72.7) | 0.623 | 0.430 |
|  |  | unclustered | 1 (0.8) | 94 (79.0) |  |  |
| CAP | *M. abscessus* | clustered | 0 (0.0) | 30 (100.0) | 1.325 | 0.250 |
|  |  | unclustered | 8 (4.3) | 180 (95.7) |  |  |
|  | *M. massiliense* | clustered | 1 (2.3) | 43 (97.7) | 0.599 | 0.439 |
|  |  | unclustered | 6 (5.0) | 113 (95.0) |  |  |
| AZM | *M. abscessus* | clustered | 26 (86.7) | 4 (13.3) | 0.088 | 0.767 |
|  |  | unclustered | 159 (84.6) | 29 (15.4) |  |  |
|  | *M. massiliense* | clustered | 41 (93.2) | 3 (6.8) | 1.975 | 0.160 |
|  |  | unclustered | 101 (84.9) | 18 (15.1) |  |  |
| LVX | *M. abscessus* | clustered | 0 (0.0) | 29 (96.7) | 0.816 | 0.366 |
|  |  | unclustered | 5 (2.7) | 177 (94.1) |  |  |
|  | *M. massiliense* | clustered | 1 (2.3) | 43 (97.7) | 0.015 | 0.904 |
|  |  | unclustered | 3 (2.5) | 112 (94.1) |  |  |
| GAT | *M. abscessus* | clustered | 9 (30.0) | 14 (46.7) | 0.791 | 0.374 |
|  |  | unclustered | 36 (19.1) | 85 (45.2) |  |  |
|  | *M. massiliense* | clustered | 4 (9.1) | 26 (59.1) | 0.009 | 0.922 |
|  |  | unclustered | 11 (9.2) | 76 (63.9) |  |  |
| TIG | *M. abscessus* | clustered | 8 (26.7) | 2 (6.7) | 2.240 | 0.135 |
|  |  | unclustered | 60 (31.9) | 48 (25.5) |  |  |
|  | *M. massiliense* | clustered | 13 (29.5) | 16 (36.4) | 0.001 | 0.972 |
|  |  | unclustered | 33 (27.7) | 40 (33.6) |  |  |
| SFX | *M. abscessus* | clustered | 17 (56.7) | 13 (43.3) | 1.495 | 0.221 |
|  |  | unclustered | 84 (44.7) | 104 (55.3) |  |  |
|  | *M. massiliense* | clustered | 11 (25.0) | 33 (75.0) | 0.096 | 0.757 |
|  |  | unclustered | 27 (22.7) | 92 (77.3) |  |  |
| STR | *M. abscessus* | clustered | 1 (3.3) | 29 (96.7) | 0.168 | 0.682 |
|  |  | unclustered | 4 (2.1) | 184 (97.9) |  |  |
|  | *M. massiliense* | clustered | 1 (2.3) | 43 (97.7) | 0.544 | 0.461 |
|  |  | unclustered | 1 (0.8) | 118 (99.2) |  |  |
| CFM | *M. abscessus* | clustered | 19 (63.3) | 11 (36.7) | 0.152 | 0.696 |
|  |  | unclustered | 112 (59.6) | 76 (40.4) |  |  |
|  | *M. massiliense* | clustered | 22 (50.0) | 22 (50.0) | 0.002 | 0.962 |
|  |  | unclustered | 60 (50.4) | 59 (49.6) |  |  |
